# Phototoxicity of Ultraviolet-A against the Whitefly *Bemisia tabaci* and Its Compatibility with an Entomopathogenic Fungus and Whitefly Parasitoid

**DOI:** 10.1155/2021/2060288

**Published:** 2021-07-10

**Authors:** Muhammad Musa Khan, Ze-Yun Fan, Dylan O'Neill Rothenberg, Jing Peng, Muhammad Hafeez, Xin-Yi Chen, Hui-Peng Pan, Jian-Hui Wu, Bao-Li Qiu

**Affiliations:** ^1^Key Laboratory of Bio-Pesticide Innovation and Application of Guangdong Province, South China Agricultural University, Guangzhou 510642, China; ^2^Engineering Research Center of Biocontrol, Ministry of Education Guangdong Province, Guangzhou 510640, China; ^3^Guangdong Laboratory for Lingnan Modern Agriculture, Guangzhou 510640, China; ^4^College of Horticulture, South China Agricultural University, Guangzhou 510642, China; ^5^State Key Laboratory Breeding Base for Zhejiang Sustainable Pest and Disease Control, Institute of Plant Protection and Microbiology, Zhejiang Academy of Agricultural Sciences, Hangzhou 310021, China

## Abstract

Ultraviolet (UV) radiation significantly affects insect life and, as a result, has been widely used to control different invertebrate pests. The current results demonstrate that when *Bemisia tabaci* first instar nymphs are exposed to UV-A light for 12, 24, 48, and 72 h, their developmental and biological parameters are negatively affected by UV-A exposure; the effect increased with an increase in exposure time. We hypothesized that UV-A light is compatible with other biological control agents. Results showed that when the entomopathogenic fungus *Cordyceps fumosorosea* was applied to third instar nymphs of *B. tabaci* previously exposed to UV-A light, the LC_50_ was 3.4% lower after 72 h of exposure to UV-A light compared to the control. However, when the fungus was exposed to UV-A light, its virulence decreased with an increase in UV-A exposure time. The parasitism rate of *Encarsia formosa* against 24 h UV-A-exposed third instar nymphs of *B. tabaci* increased while the adult emergence from parasitized nymphs was not affected after UV-A light exposure. Parasitism rate was significantly reduced however following *E. formosa* exposure to UV-A light; but again, adult emergence was not affected from parasitized nymphs. The percentage mortality of *E. formosa* increased with increasing exposure time to UV-A light. The enzyme activity of SOD, CAT, GST, and AChE and the energy reserve contents were negatively affected due to UV-A exposure. Collectively, this study has demonstrated that UV-A light significantly suppresses the immune system of *B. tabaci* and that UV-A light is compatible with other biological control agents if it is applied separately from the biological agent.

## 1. Introduction

Climate change can have various effects on abiotic stress. As a result, it poses a threat to agricultural systems' long-term viability and productivity. Stress factors, both abiotic and biotic, also play an important role in the life of an insect. There are numerous environmental stresses, such as ozone depletion, increased ultraviolet irradiation, biodiversity destruction, and temperature that impact insects. For many organisms, ultraviolet (UV) radiation is a significant source of stress [1]. UV radiation is currently attracting much attention due to its potentially harmful effects on a variety of species. UV radiation is generally considered a resilient ecological stressor for biological entities [2]. It may damage nucleic acids, membrane fatty acids, and various amino acids [3, 4], resulting in cell toxicity, genetic modifications, and cell signaling pathway modifications [5]. UV exposure shortens an insects' adult lifespan, increases oviposition rate, and reduces fertility [6, 7]. UV causes the development of reactive oxygen species (ROS), which can damage nucleic acids, membrane lipids, and proteins [4]. These various types of molecule damage have significant biological consequences, such as genetic variation, cytotoxicity, and signaling cell pathway transformation [5].

The whitefly, *Bemisia tabaci*, is a key pest of many greenhouse vegetables, ornamentals, and also field crops worldwide since the midseventies [8]. This whitefly is polyphagous and leads to serious economic losses [9]. The damage to crops is manifold; the feeding of *B. tabaci* causes direct damage to plant foliage. In China, for example, *B. tabaci* MEAM1 is well distributed across 31 provinces or municipalities and causes significant economic losses [10]. Adult and immature whitefly are phloem feeders [11]. Furthermore, *B. tabaci* acts as a vector of around 150 plant viruses [12].

Insecticides have been the mainstay of controlling *B. tabaci* in diverse agricultural production systems. Resistance to insecticides resulting in loss of efficacy of many older insecticides has placed excessive pressure on novel products. Alternative control strategies now have a key role in controlling *B. tabaci* and managing pesticide resistance development. Using biological agents is not only environmentally safe but can also have a significant impact on whitefly management practices. To date, more than 20 species of entomopathogenic fungi are known to infect whitefly. Among these, the entomopathogenic fungus, *Cordyceps fumosorosea* Zare and Gams (Hyphomycetes), formerly known as *Isaria fumosorosea*, designated as Cordyceps clade [13], is a well-known fungal species used for whitefly management and has been widely studied [14–17]. *C. fumosorosea*-based formulations have been commercially available since the 1990s for the management of whitefly [18, 19]. *Encarsia formosa* Gahan (Hymenoptera: Aphelinidae) also plays an important role in managing *B. tabaci* [20, 21]. *E. formosa* is a solitary endoparasitoid that is commercially used as a biocontrol agent for *B. tabaci* [22, 23]. *E. formosa* kills 75% of its whitefly host by probing nymphs with its ovipositor and depositing eggs in their bodies. Larvae of *E. formosa* then feed on the parasitized whitefly internal contents, eating all the organs and leaving only the outer armor in which they pupate from afterwards [24].

UV radiation and other abiotic stressors have a major impact on insect life because they increase the development and accumulation of reactive oxygen species (ROS). These oxygen free radicals increase both the antioxidant potential and oxidant development of cells. They are not harmful at low concentrations and play important roles in cell signaling and defense [25–27]. Multiple biochemical pathways contain carbohydrates, proteins, and lipids as an end product in the energy metabolism of insects [28]. Different physiological processes such as synaptic transmission, morphogenetic behaviors, phospholipid synthesis, sexual maturation, and egg development may be influenced by these carbohydrates, proteins, and lipids (which serve as insect primary energy sources) [29, 30]. As both biological control agents and insect pests share a common habitat [31], it is obvious that the application of UV light has the potential to affect other natural whitefly controlling measures.

The current study was designed to examine the effectiveness of UV-A irradiation against *B. tabaci* by evaluating both biological and physiological parameters. The risks posed to the entomopathogenic fungus and the whitefly parasitoid by UV-A light were also assessed by evaluating the pathogenicity and percentage parasitism, respectively.

## 2. Materials and Methods

### 2.1. Insects, Plants, and Entomopathogenic Fungus

*Gossypium hirsutum* L. (cotton plants) were raised under glasshouse conditions at South China Agricultural University (SCAU), Guangzhou, in 15 cm diameter plastic pots containing a mixture of soil consisting of 5% clay, 85% peat, and 10% sand to reach the 7–8 expanding leaf stage. Two symmetrical, completely expanded leaves of identical size were used in all experimental replications.

The silverleaf whitefly, *B. tabaci* Middle East-Asia Minor 1 (formerly known as *B. tabaci* B biotype), was the *B. tabaci* species used in this study. Different cotton leaves were caged, and 60 pairs of the whitefly were released into the cage for egg-laying to occur for 24 hours. The adults of the whitefly were then removed after 24 hours, and the plants were kept at a temperature of 26 ± 1°C, relative humidity of 60%, and photoperiod of 14 : 10 (L : D) in an iron-framed and plastic sieve cage (60 × 60 × 60 cm) to allow for whitefly development as outlined by Ou et al. [32] (Figure S1).

In 2015, *E. formosa* was obtained from the Chinese Academy of Agricultural Sciences, Institute of Plant Protection. Under laboratory conditions at SCAU, the parasitoid population was reared at the temperature of 26 ± 1°C, relative humidity of 60%, and a photoperiod of 14 : 10 (L : D) on cotton plants containing third instar whitefly nymphs placed in an iron-framed and plastic sieve cage (60 × 60 × 60 cm) as outlined by Ou et al. [32].

*C. fumosorosea* isolate SP535 was originally isolated from soil obtained from the repository of the Key Laboratory of Biopesticides Innovation and Application of Guangdong Province, SCAU. Potato Dextrose Agar (PDA) media was used to maintain the fungal culture (200 g potatoes, 20 g dextrose, and 20 g agar in 1 L of distilled water) in a glass dish (90 mm Ø) for three weeks under dark conditions at 25 ± 2°C. The methodology followed for maintaining the experimental materials was as outlined by Ou et al. [32].

### 2.2. UV-A Irradiation Source

A UV-A irradiation source of 15 W power with 360 nm wavelength was used during the experimentation. The UV-A light source was purchased from Shenzhen Guanhongya Photoelectronic Technology Co. Ltd. (Shenzhen, China).

### 2.3. Effect of UV-A Light on Whitefly Development and Biology

To assess the effect of UV-A on the developmental period and other life table parameters of *B. tabaci*, first instar nymphs of *B. tabaci* were used. To obtain a homogeneous first instar nymphal population, cotton plants at the 6-8 expanded leaf stage were used. Leaves were caged, and whitefly adults were released for egg-laying for 24 hours. After removing the adult whiteflies, the plants were kept at 26 ± 1°C and 16 : 8 (L : D) photoperiod for 5-7 days. After the emergence of 80% of first instar nymphs, they were then exposed to UV-A light. A plant containing first instar nymphs was kept under dark conditions before being exposed to UV-A light. The plants containing the first instar nymphs of *B. tabaci* were exposed to UV-A light for 12, 24, 48, and 72 hours. Similar infested plants which were not exposed to UV light acted as controls. The distance between the UV-light source and the leaf containing the first instar nymphs of *B. tabaci* was approximately 50 cm. After exposure of 12, 24, 48, and 72 hours, the plants were exposed to normal light. As nymphs reached the second instar, 90 of them were marked on a leaf with each nymph being given a specific number to aid identification. Each marked nymph was taken as a single replication. Individuals were observed each day, and their current instar was recorded until the nymph either developed into an adult or died. Each emerged female was paired with an emerged male from the same treatment and kept in a small petri dish (3 cm) containing agar gel and a (2 cm) cotton leaf disk. The petri dish was observed daily to calculate fecundity and the number of days of female longevity. A new leaf disk was provided daily to avoid the risk of starvation. The remaining males were kept in a petri dish with agar and cotton leaf disk separately to assess their longevity.

### 2.4. Effect of UV-A Light on Enzyme Activity and Energy Reserves

*B. tabaci* adults were exposed to UV-A light for 0 (control), 12, 24, 48, and 72 hours. One hundred and fifty adults per treatment per replication were collected. Three technical and three biological replications were established. Collected samples were weighed before homogenization. The total protein content of supernatants from the insect homogenates was determined using bovine albumin serum (BSA) as a norm, as described by Nazir et al. [33].

Adult *B. tabaci* was homogenized with ice-cold 0.05 M sodium phosphate buffer at room temperature (pH 7.3). At 4°C, the homogenized samples were centrifuged for 10 minutes at 12,000 rpm (rotation per minute). The supernatant was collected and moved to new tubes and centrifuged for 15 minutes at 4°C at 12,000 rpm. For the various enzyme assays, the final supernatants were used for enzyme preparation. Antioxidant enzyme (SOD, POD, CAT, and PPO) and detoxification enzyme (AChE, GST, and P450) activity was determined by the commercially available kits purchased from Nanjing Jiancheng Bioengineering Research Institute. The method followed was as per the instructions provided by the manufacturer.

To estimate the whitefly energy reserve contents after exposure to UV-A light, adults were again exposed to UV-A light as outlined above, and samples were collected. The whole bodies of adult *B. tabaci* were homogenized in sodium phosphate buffer pH 7.0. The samples were then centrifuged at 4°C for 10 minutes at 10,000 rpm. The collected supernatants were used in further experimentation. For the estimation of energy reserves, commercially available kits to determine cholesterol, glycogen, and triglyceride were purchased from Solarbio Life Science, Beijing, China. The method followed was as per the instructions provided by the manufacturer.

### 2.5. Effect of UV-A Light on the Virulence of *C. fumosorosea* against *B. tabaci*

To determine the virulence of *C. fumosorosea* against *B. tabaci*, the progressive concentrations were prepared until the mortality ranged between 10 and 95%. The leaf dip application method was used as outlined by Nazir et al. [33]. In a 250 mL reagent container, a stock suspension of conidia was made containing 100 mL distilled water and 0.05% (*v*/*v*) Tween 80® by adding the mass sporulating culture. The mixture was shaken vigorously to isolate spores from the hyphal debris. The conidial concentration was determined using an improved Neubauer hemocytometer (Brand GmbH, Wertheim, Germany). The conidial suspensions were diluted in sterilized water containing 0.05% Tween 80 in five separate suspensions (i.e., 1 × 10^8^, 1 × 10^7^, 1 × 10^6^, 1 × 10^5^, and 1 × 10^4^ conidia mL^−1^). Distilled water containing Tween 80 at 0.05% was used as control. Plants at 7-8 extended leaf stage containing third instar nymphs were used in this experiment. A germination test was performed on a PDA medium before performing bioassays against the whitefly to determine the percentage of viable conidia [34]. Conidial germination was greater than 95% in all bioassays. Plants containing third instar nymphs were kept in the dark for 2 hours and then exposed for 0 (control), 12, 24, 48, and 72 hours to UV-A light. Leaves were dipped into conidial suspensions for 10 seconds and then air-dried on tissue paper for 15 min at room temperature. Each leaf contained 20 third instar nymphs per treatment per replication. Whitefly mortality was recorded after five days of fungal application. The leaves were plucked and placed on Petri plates containing agar gel to maintain leaf moisture and were kept under dark conditions at relative humidity of 90% to stimulate fungal growth to confirm the whitefly mortality due to fungal infection. Percentage mortality was calculated by using the Abbott formula [35].

To assess the effect of UV-A light on the fungus, the fungal culture was exposed to UV-A light for 12, 24, 48, and 72 h. The fungal culture plates were placed in an environmental chamber under dark conditions, and an external UV-A light source was used to expose the fungi. After exposure, treatment with the conidial suspension was undertaken by the method outlined above.

### 2.6. Effect of UV-A Light on Parasitism of *E. formosa* against *B. tabaci*

To determine the effect of UV-A light on the parasitism of *E. formosa*, cotton plants containing third instar nymphs of *B. tabaci* (<24 h old) were prepared as outlined above. This experiment was divided into two different parts: (1) whitefly was exposed to UV-A light, and then, *E. formosa* was released; and (2) *E. formosa* was exposed to UV-A light and then released onto the whitefly nymphs.

For the first part, cotton plants containing third instar nymphs of whitefly were exposed to UV-A light for 0 (control), 12, 24, 48, and 72 h. Two hundred third instar nymphs (<24 h old) were kept on the leaf, and the remaining nymphs were removed from the leaf using a camel hairbrush. Leaves were caged as shown in Figure S1, and the unexposed parasitoid (<24 h old) with a ratio of 1 : 20 (parasitoid : nymphs) was released into the cages for 24 hours. Each treatment was replicated three times. The number of parasitized nymphs was recorded after ten days. The nymphs that turned brown were considered parasitized. Percentage emergence was then determined by caging the leaves for more than five days. After five days, cages were removed, and the number of emerged adults of *E. formosa* was counted and recorded. Percentage emergence was determined by using the following formula:

(1) $$\begin{array}{c} \text{Percentage Emergence}=\frac{\text{Number of parasitoids emerged from parasitized nymphs}}{\text{Total number of parasitized nymphs}}\times 100. \end{array}$$

For the second part, the same procedure as outlined above was followed except with the difference that *E. formosa* (<24 h old) were exposed to UV-A light for 0 (control), 12, 24, 48, and 72 h and then released onto third instar nymphs of *B. tabaci*.

### 2.7. Statistical Analysis

The development time of different stages, the survival rate of different stages, the fecundity off, and female whitefly preoviposition duration and adult longevity were all studied using the age-stage two-sex life table model [36, 37]. TWOSEX-MS Chart program was downloaded from the website http://140.120.197.173/Ecology/prod02.htm [38]. Using 100,000 bootstrap replicates, standard errors (SE) and means were measured [40, 41]. In the TWOSEX-MS Chart, the paired bootstrap method was used to compare all treatments [39].

The software PoloPlus (Version: 1.0 (Pacific Southwest Forest and Range Experiment Station, Berkeley, California, USA)) was used to calculate LC_50_ of *C. fumosorosea* against *B. tabaci*. The data of mortality in replications were subjected to test the hypothesis of equality and parallelism via PoloPlus by following the method described by Chang and He [41]. To analyze the parasitism rate among the treatments and enzymatic activity, one-way ANOVA, along with the Tukey post hoc test at *P* < 0.05, was used to compare the means via SPSS. SigmaPlot 12.0 was used to form graphical work for all parameters. Correlation of enzymatic activity and energy reserves with exposure time to UV-A light was calculated via RStudio v1.3.1056 software with the corrplot package.

## 3. Results

### 3.1. Effect of UV-A Light on the Development, Growth, and Dimorphic Parameters of *B. tabaci*

The effect of UV-A light on the development period of the preadult stages of *B. tabaci* is shown in Table 1. The presented results demonstrate that the developmental period of the first, second, third, and fourth instars was significantly reduced with increasing UV-A exposure time. The male and female longevity was also reduced at 24, 48, and 72 hours of exposure compared to controls. The APOP (adult preoviposition) was not significantly affected by the UV-A light exposure; the TPOP (total preoviposition period) was significantly reduced at 48 and 72 hours of exposure to UV-A light as compared to the control. Following 72 hours of UV-A exposure also significantly reduced the total fecundity of *B. tabaci*.

Similarly, results also showed that the fecundity was significantly reduced with the increasing trend of UV-A light exposure compared to the control (Table 2). The effect of UV-A light on the population growth of *B. tabaci* is given in (Table S1). Results show that *r* (intrinsic rate of increase) and *λ* (finite rate of increase) were not affected due to UV-A light exposure. At the same time, *R*_0_ (net reproductive rate) was negatively affected by UV-A light exposure after 48 and 72 h. More exposure time lessened the net reproductive rate as compared to the control. The mean generation time was significantly reduced due to UV-A light exposure after 72 hours compared to the control.

The *S*_*xj*_ (survival rate of the specific stage) value was reduced in UV-A-exposed *B. tabaci*. The survival rate of females and males in controls was observed to be 35 and 33 days, 33 and 36 days after 12 hours of exposure, 34 and 34 days after 24 hours of exposure, 30 and 33 days after 48 hours of exposure, and 27 and 33 days after 72 hours of exposure, respectively. Results showed that UV-A light exposure significantly reduced male and female survival rates (Figure S2).

Results for *l*_*x*_ (survival rate of the specific stage), *f*_*x*_ (fecundity of specific age stage), *m*_*x*_ (overall population fecundity), and *l*_*x*_*m*_*x*_ (total maternity) are shown in Figure S3. The *S*_*xj*_ curves are expressed by *l*_*x*_, which is a simple form of the *S*_*xj*_ curves. The results showed that as UV-A light exposure time increased, the survival rate decreased, affecting other reproductive parameters. As the survival curve started to reduce with time, *f*_*x*_, *m*_*x*_, and *l*_*x*_*m*_*x*_ also reduced. In addition, the fecundity value was also reduced in line with a reducing survival rate.

Results of *E*_*xj*_ showed that due to UV-A light exposure, the female life expectancy was significantly reduced compared to male life expectancy. In the controls, the life expectancy of females and males was observed at 36.04 and 34 days, respectively. When nymphs were exposed to UV-A light, the male life expectancy after 12, 24, 48, and 72 hours was 34.00, 32.87, 33.89, and 32.99 days, respectively, which was not significantly altered following UV-A exposure. In comparison, a significant reduction in female life expectancy following 12, 24, 48, and 72 hours of exposure was observed: 35.92, 34.00, 30.06, and 27.01 days, respectively (Figure S4).

Results of *V*_*xj*_ (reproduction of a specific stage) show how each individual fits into the next population. Results (Figure S5) showed that on the 25^th^ day, the maximum female reproductive value (106.85) was observed. After exposure to UV-A light for 12, 24, 48, and 72 hours, the reproductive value *V*_*xj*_ was 84.39 on the 19^th^ day, 72.43 on the 19^th^ day, 60.99 on the 18^th^ day, and 52.69 on the 15^th^ day, respectively. Results showed that UV-A light exposure not only reduced the duration of reproduction but also reduced age-specific reproduction.

### 3.2. Effect of UV-A Light Exposure on the Enzyme Activity and Energy Reserve Contents of *B. tabaci*

**Figure 1:**
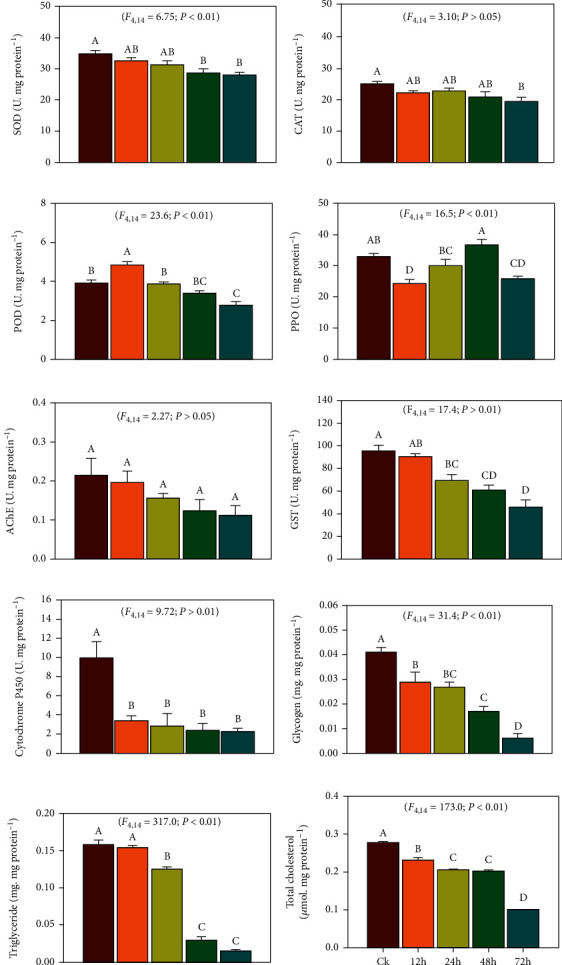
The activity of different enzymes (a) SOD, (b) CAT, (c) POD, (d) PPO, (e) AChE, (f) GST, and (g) cytochrome P450 and contents of different energy reserves (h) glycogen, (i) triglyceride, and (j) total cholesterol of *Bemisia tabaci* exposed to UV-A light for control (0 h), 12 h, 24 h, 48 h, and 72 hours. The bars are showing the mean value of three replications. Standard error bars are showing the standard deviation of the mean. Lowercase lettering is showing the significance among the treatments at *P* < 0.05. Similar letters have no significant difference among the treatments.

The enzymatic analysis showed that following exposure to UV-A light, a significant reduction in enzyme activity was observed in the exposed treatments compared to the control. The activity of SOD, POD, PPO, GST, and cytochrome P450 and contents of glycogen, triglyceride, and total cholesterol were continuously reduced as the UV-A light exposure time increased. At the same time, no significant effect was observed in CAT and AChE enzyme activity (Figures 1).

**Figure 2:**
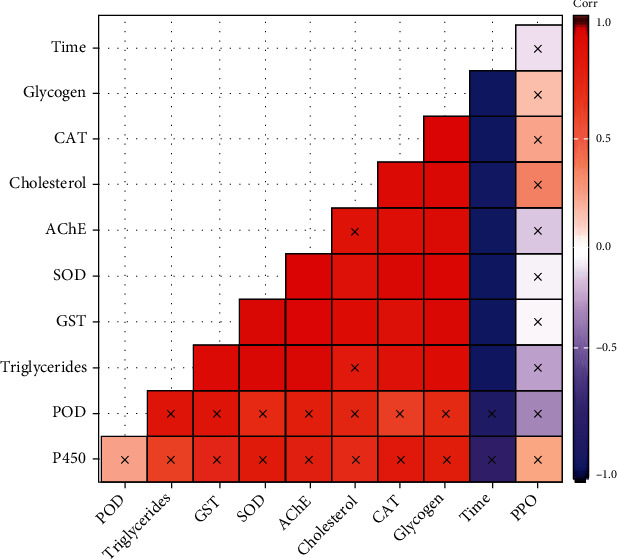
Correlation matrix of UV-A light exposure time and enzyme activity and energy parameters of *Bemisia tabaci*. The intensity of color shows the correlation's strength, while the cross sign shows a nonsignificant correlation at *P* < 0.05.

Results of correlation analysis (Figure 2) of different oxidative and detoxification enzyme activity and energy reserves with UV-A light exposure time demonstrated that, except for PPO, POD, and P450 enzyme activity, all other enzymes had a significant negative correlation with exposure time. These results showed that UV-A light exposure caused depletion of different enzyme activity as the exposure time increased.

### 3.3. Effect of UV-A Light on the Virulence of *C. fumosorosea* against *B. tabaci*

The results of the virulence of *C. fumosorosea* against *B. tabaci* are shown in Table 3. The virulence assessment bioassay showed that LC_50_ in the control (without UV-A exposure) was 2.1 × 10^5^ conidia mL^−1^ (4.5 × 10^4^-6.7 × 10^5^; *χ*^2^ = 1.69; *P* = 0.564). As the exposure time of UV-A light increased, the LC_50_ concentration decreased. The maximum percentage mortality was recorded in the treatment where 1 × 10^8^ conidial suspension was applied onto 72 hours of UV-A light-exposed nymphs.

When the fungus was exposed to UV-A light for different time periods, the results showed that with an increase in exposure time, the LC_50_ also increased. The virulence assessment showed that LC_50_ in the control (without UV-A exposure) was 2.3 × 10^6^ and after 72 hours of exposure was 5.9 × 10^8^. The maximum percentage mortality was observed where third instar nymphs of *B. tabaci* were treated with 1 × 10^8^ conidial suspension exposed to UV-A light for 12 hours.

### 3.4. Effect of UV-A Light on Parasitism of *E. formosa* against *B. tabaci*

**Figure 3:**
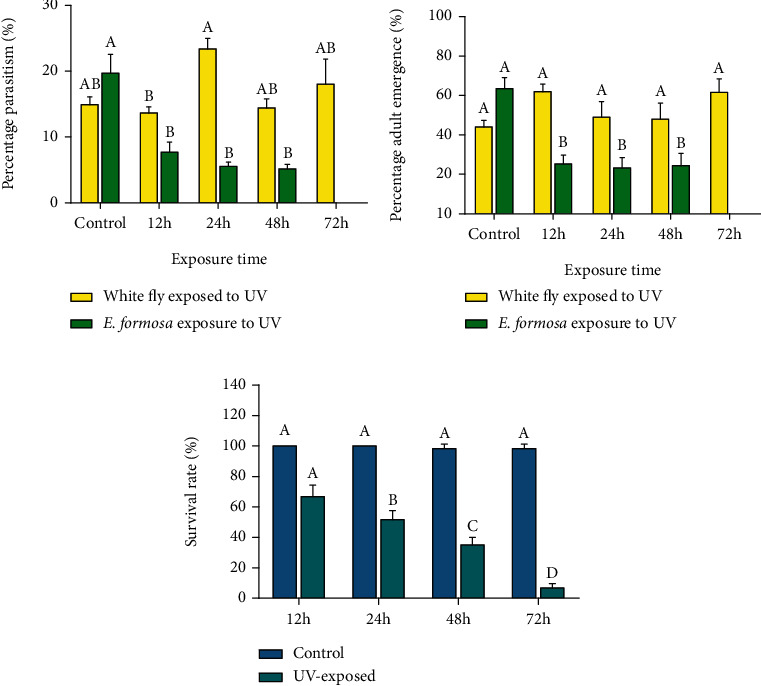
(a) Percentage parasitism, (b) percentage adult emergence, and (c) percentage survival of *Encarsia formosa* exposed to UV-A light for control (0 h), 12 h, 24 h, 48 h, and 72 h. Bars are showing the mean of three replications. Error bars are showing the mean deviation. Lowercase lettering is showing statistically significant differences among different treatments (*P* < 0.05). Similar lettering has no significant difference. Error bars are showing mean deviation.

*B. tabaci* third instar nymphs firstly exposed to UV-A light and then exposed to *E. formosa* to assess percentage parasitism showed (Figure 3) that the nymphs exposed 24 hours to UV-A light were parasitized more than the 12, 48, and 72 hours of exposures (*F*_4,14_ = 3.82; *P* < 0.05). The results showed that UV-A light weakened the nymphs of *B. tabaci* so aiding *E. formosa* to parasitize more. The resulting percentage emergence was not significantly affected. However, when *E. formosa* was exposed to UV-A light, its parasitic ability was significantly reduced compared to the control (*F*_4,14_ = 15.2; *P* < 0.01) (Figure 3(a)). The percentage of adult emergence of *E. formosa* emerging from parasitized nymphs was the same in the control and UV-A light-exposed nymphs (*F*_4,14_ = 1.71; *P* > 0.05). In addition, the percentage of adult emergence was also significantly reduced when the parasitoid was exposed to UV-A light as compared to that in the control (*F*_4,14_ = 13.3; *P* > 0.05) (Figure 3(b)). During the experimentation, it was observed that *E. formosa* is sensitive to UV-A light. A significant number of *E. formosa* adults exposed to UV-A light simply died. So, to assess the survival rate of *E. formosa*, adults were exposed to UV-A light for 12, 24, 48, and 72 hours. Results showed that as the UV-A exposure time increased, the survival percentage significantly decreased, which showed that UV-A light is lethal for *E. formosa* and so both could not be used together to control *B. tabaci* (Figure 3(c)).

**Figure 4:**
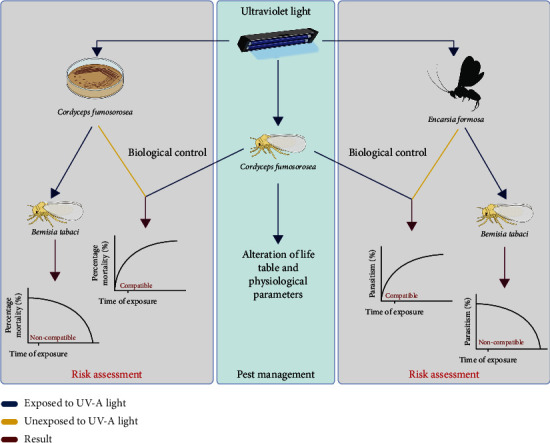
Schematic diagram of a model using UV-A light as a tool to control *Bemisia tabaci* and compatibility with other biological control agents.

All the results provide a model that demonstrates UV-A light can be an effective tool to control *B. tabaci*. UV-A light and biological control agents of *B. tabaci* could be used together if the UV-A light is applied to the whiteflies before applying the biological control agent. Exposure to UV-A light seriously affected the effectiveness of the biological control agents investigated (Figure 4).

## 4. Discussion

The current study has demonstrated that due to UV-A light exposure, the nymphal developmental period, preoviposition period, fecundity, female longevity, and other dimorphic parameters of *B. tabaci* were significantly affected. Results also showed that survival rate, reproduction rate, and life expectancy also reduced with increased UV-A exposure duration. Our present results coincide with Ali et al. [42] who reported that due to UV-A light exposure, the developmental period of immature stages of *Mythimna separata* (eggs, larvae, and pupa) declined with increased exposure time. The male and female longevity was also reported to decrease with an increase in exposure time. Similarly, Tariq et al. [23] experimented by exposing *Dialeurodes citri* to UV-A light and reported a significantly shorter developmental time of *D. citri* immatures (nymphs and pupa) in the F1 generation after adults were exposed to UV-A light. However, both male and female longevity was significantly reduced with exposure time. Zhang et al. [7] also reported an increase in the immature development period of *Helicoverpa armigera* along with a decrease in male and female longevity as UV exposure time increased. All the stated results confirm that UV-A exposure can significantly affect an insect's developmental period, but the cause of this change in the developmental period is still unknown.

Different antioxidants are in a complex equilibrium state within an insect species in normal circumstances in order to maintain normal physiological activities. Insects have also developed a complex network of antioxidant enzyme systems [43] and detoxification enzymes [44] to fight against the risk of oxidative stress. In the current study, we observed that due to UV-A light exposure, SOD, POD, and PPO activity was significantly affected. Moreover, the activity of detoxification enzymes was also affected due to UV-A exposure. Many previous studies reported the alteration of enzyme activity when exposed to UV irradiation. Ali et al. [45] reported that UV-A exposure caused oxidative stress in *M. separata* and altered antioxidant enzymes' activity. Zhou et al. [46] exposed grain aphid *Sitobion avenae* to UV-B radiation and reported that the antioxidant enzymes SOD, POD, and CAT were significantly affected. UV exposure resulted in more than 90% reduction in PPO activity after 5 minutes of exposure [47]; when *Dendrolimus tabulaeformis* were exposed to UV-A light, elevated oxidative stress and disturbed physiological detoxification were recorded [44]. The current study showed that due to UV-A light exposure, a significant decrease in the contents of energy-providing compounds like glycogen, triglyceride, and total cholesterol was observed. It is a basic life history theory that reproduction always costs survival [48]. These two processes share the energy to maintain normal biological function under UV stress [42]. After exposure to UV light, a reduction in these energy reserves justifies a reduction in reproduction and longevity of *B. tabaci*. However, there is no study available to justify reducing energy reserves due to UV-light exposure; only Ohkawara et al. [49, 50] reported reducing glycogen in humans and minipigs due to UV exposure.

A previous study showed that *C. fumosorosea* (formerly *I. fumosorosea*) is an effective biological control agent against *B. tabaci*, not only being a mycoinsecticide but also in that it exerts oxidative stress in *B. tabaci* [51]. Santos et al., Wang et al., and Zou et al. [52–54] described that *C. fumosorosea* is a very efficient and environmentally safe control agent for *B. tabaci.* However, exposure to solar radiation can strongly affect conidial development, survival, dispersion, dissemination, germination, pathogenesis, and virulence, although its effects often vary from species to species [55–64].

The current study showed that due to UV-A light exposure to *B. tabaci*, the parasitism rate by *E. formosa* was increased, but the percentage emergence of parasitoids was not affected. Cochard et al. [65] exposed *Aphidius ervi* to UV and reported that due to UV-light exposure, a significant change in parasitism by *A. ervi* was noted, but adult emergence remained unaffected. Our results also showed that UV-A light exposure decreased the parasitism of *E. formosa* and also caused mortality in *E. formosa*. It has been reported that UV-light exposure significantly reduced the parasitism of *Trichogramma cacoeciae*, an egg parasitoid of *Lobesia botrana* [66]. Indeed, many studies report the toxicity of UV light against different insects [67–71]. Due to cascading effects in the food chain, insects higher up in the food chain are thought to be affected more by environmental stress than those at lower levels. As a result, a change in parasitism rate could be described as an instant stress response [72–74]. The factors that influence parasitoid species composition are difficult to identify, and the reasons why some species react to stress more effectively than others have remained unclear [75].

Due to exposure to high-intensity light or UV light, plants usually respond by producing ROS (reactive oxygen species), which has already been reported as a defense against diseases and pests [76]. The abrupt accumulation of hydrogen peroxide on the pathogen target site makes it toxic for pathogens [77]. ROS is also involved in triggering signaling pathways responsible for the activation of defense mechanisms, for example, the production of secondary metabolites, which are defense compounds [77–79]. In addition, Ouhibi et al. [80] has recently shown that after UV-C light exposure against *Botrytis cinerea* and *Sclerotinia minor*, the increased resistance that was observed may include phenolic compounds. It could also be speculated that phytoalexins' biosynthesis can be due to the increased resistance to UV-C treatments [81, 82]. In UV-C-treated tomato fruit, higher glycoalkaloid alpha-tomatine levels, an antifungal compound, showed resistance against *Rhizopus stolonifer* [83]. UV-induced resistance to fungi of the genus Penicillium was associated with the accumulation of scoparone and scopoletin phytoalexins in citrus fruits and structural barriers [84]. However, some studies have reported that UV light has a negative impact on the plant's morphology and physiology; Kakani et al. [85] reported that UV-B light reduced plant height, branch length, leaf area, flower and petal length, petal area, and wax content. However, no reduction in production has been reported regarding exposure to UV light.

## 5. Conclusion

From the current study, it can be concluded that UV-A light can be used as a *B. tabaci* management tool. UV-A light affects the adults' development duration, longevity, and reproduction as well as affecting its physiology by disrupting the enzymatic balance. The UV-A light exposure exerted stress in the *B. tabaci* and suppressed the immune system, which gave an opportunity to the entomopathogenic fungus to work more efficiently. Therefore, due to the suppression of the immune system, the biological control agent can significantly control *B. tabaci* better. UV-A light can only be applied before the application of the biological control agent. According to the known literature and current experimental trials, both the entomopathogenic fungus and the parasitoid are sensitive to UV-A application. The literature also documents that UV-light exposure helps plants induce resistance, but no effect on productivity has been recorded. This study has laid the basis for conducting investigations on the application of UV-A light for the management of *B. tabaci* under semifield or greenhouse conditions.

## Figures and Tables

**Table 1:** Effect of UV-A light exposure on the developmental period (mean ± SE) of preadult stages of whitefly *Bemisia tabaci.*

Treatments	The development period of immature stages			
	1^st^ instar (d)	2^nd^ instar (d)	3^rd^ instar (d)	4^th^ instar (d)
Control	5.8 ± 0.1^a^	2.3 ± 0.1^a^	3.1 ± 0.1^a^	2.7 ± 0.1^a^
12 hours	5.4 ± 0.1^ab^	2.3 ± 0.1^ab^	2.9 ± 0.1^ab^	2.1 ± 0.1^ab^
24 hours	5.3 ± 0.1^b^	2.0 ± 0.1^bc^	2.8 ± 0.1^ab^	1.9 ± 0.1^b^
48 hours	5.2 ± 0.1^bc^	1.9 ± 0.1^c^	2.6 ± 0.1^b^	1.8 ± 0.1^b^
72 hours	4.8 ± 0.1^c^	1.7 ± 0.1^c^	2.6 ± 0.1^b^	1.9 ± 0.1^b^

The same lowercase letters in the same column are not significantly different based on the paired bootstrap test at the 5% significance level. Ninety nymphs were used for each treatment. d = days.

**Table 2:** Effect of UV-A light exposure on the life parameters (mean ± SE) of *Bemisia tabaci* adults exposed at the nymphal stage.

Treatments	Female adult longevity (d)	Male adult longevity (d)	APOP (d)	TPOP (d)	Fecundity (eggs/female)
Control	33.0 ± 0.5^a^	30.4 ± 0.4^a^	1.7 ± 0.1^a^	19.6 ± 1.2^a^	217.0 ± 14.7^a^
12 hours	31.0 ± 0.4^a^	29.1 ± 0.4^a^	1.6 ± 0.1^a^	17.9 ± 0.4^b^	180.9 ± 9.3^b^
24 hours	29.4 ± 0.4^b^	26.9 ± 0.6^b^	2.1 ± 0.1^a^	18.6 ± 0.3^a^	134.5 ± 9.7^c^
48 hours	26.8 ± 0.5^b^	25.7 ± 0.6^b^	2.1 ± 0.2^a^	17.4 ± 0.4^b^	105.5 ± 9.9^d^
72 hours	23.8 ± 0.6^c^	23.5 ± 0.4^c^	1.8 ± 0.2^a^	16.8 ± 0.3^c^	87.26 ± 7.2^e^

The same lowercase letters in the same column are not significantly different based on the paired bootstrap test at the 5% significance level. Ninety nymphs were used for each treatment. d = days.

**Table 3:** LC_50_ of *Cordyceps fumosorosea* against *Bemisia tabaci.*

	Treatment	LC_50_	Limit (95% C.I.)	Slope ± SE	Chi-square	*P* value (df)
*B. tabaci* exposed to UV-A light	Control	2.1 × 10^5^	4.5 × 10^4^‐6.7 × 10^5^	0.302 ± 0.06	1.69	0.56 (3)
	12 hours	3.4 × 10^4^	9.4 × 10^3^‐8.4 × 10^4^	0.466 ± 0.06	2.94	0.98 (3)
	24 hours	3.3 × 10^4^	7.7 × 10^3^‐8.7 × 10^4^	0.425 ± 0.06	1.67	0.56 (3)
	48 hours	9.4 × 10^3^	1.2 × 10^3^‐3.3 × 10^4^	0.410 ± 0.07	0.98	0.34 (3)
	72 hours	7.3 × 10^3^	1.3 × 10^3^‐2.1 × 10^4^	0.508 ± 0.08	1.79	0.60 (3)

*C. fumosorosea* exposed to UV-A light	Control	2.3 × 10^6^	7.6 × 10^5^‐6.2 × 10^6^	0.392 ± 0.06	1.43	0.47 (3)
	12 hours	1.2 × 10^7^	4.6 × 10^6^‐3.3 × 10^7^	0.371 ± 0.06	0.81	0.27 (3)
	24 hours	5.6 × 10^6^	9.7 × 10^5^‐2.5 × 10^7^	0.237 ± 0.05	2.49	0.83 (3)
	48 hours	3.6 × 10^7^	6.8 × 10^5^‐4.4 × 10^8^	0.210 ± 0.05	0.43	0.14 (3)
	72 hours	5.9 × 10^8^	9.4 × 10^7^‐4.4 × 10^10^	0.225 ± 0.06	0.40	0.13 (3)

SE = standard deviation of the slope; df = degree of freedom; C.I. = confidence.

## Data Availability

All the data has already been given in the manuscript and supplementary material.

## Supplementary Materials

Supplementary Materials Figure S1: caging of cotton leaves during whitefly rearing and experimentation. Figure S2: graphs show *S*_*xj*_ (survival rate of the specific stage) of *Bemisia tabaci* treated at second instar nymph stage exposed to UV-A light. Figure S3: graphs show *l*_*x*_ (survival rate of the specific stage), *f*_*x*_ (fecundity of specific age stage), *m*_*x*_ (overall population fecundity), and *l*_*x*_*m*_*x*_ (total maternity) values for *Bemisia tabaci* nymphs exposed to UV-A light. Figure S4: graphs show *E*_*xj*_ (life expectancy) values of *Bemisia tabaci* nymphs exposed to UV-A light. Figure S5: graphs show *V*_*xj*_ (reproduction of a specific stage) values for *B. tabaci* nymphs exposed to UV-A light. Table S1: effect of UV-A light exposure on the population growth parameters (mean ± SE) of *Bemisia tabaci* adults exposed at the nymphal stage. Table S2: percentage mortality of *Bemisia tabaci* exposed to UV-A light for the different durations and treated with different concentrations of *Cordyceps fumosorosea.*
