# Supplementary material for: Phototoxicity of Ultraviolet-A against the Whitefly Bemisia tabaci and Its Compatibility with an Entomopathogenic Fungus and Whitefly Parasitoid
Source: Oxid Med Cell Longev. 2021 Jul 10;2021:2060288. doi: 10.1155/2021/2060288 (PMC8289603; doi:10.1155/2021/2060288)
Supplement: Supplementary Materials — Figure S1: caging of cotton leaves during whitefly rearing and experimentation. Figure S2: graphs show Sxj (survival rate of the specific stage) of Bemisia tabaci treated at second instar nymph stage exposed to UV-A light. Figure S3: graphs show lx (survival rate of the specific stage), fx (fecundity of specific age stage), mx (overall population fecundity), and lxmx (total maternity) values for Bemisia tabaci nymphs exposed to UV-A light. Figure S4: graphs show Exj (life expectancy) values of Bemisia tabaci nymphs exposed to UV-A light. Figure S5: graphs show Vxj (reproduction of a specific stage) values for B. tabaci nymphs exposed to UV-A light. Table S1: effect of UV-A light exposure on the population growth parameters (mean ± SE) of Bemisia tabaci adults exposed at the nymphal stage. Table S2: percentage mortality of Bemisia tabaci exposed to UV-A light for the different durations and treated with different concentrations of Cordyceps fumosorosea. [file 2060288.f1.doc]

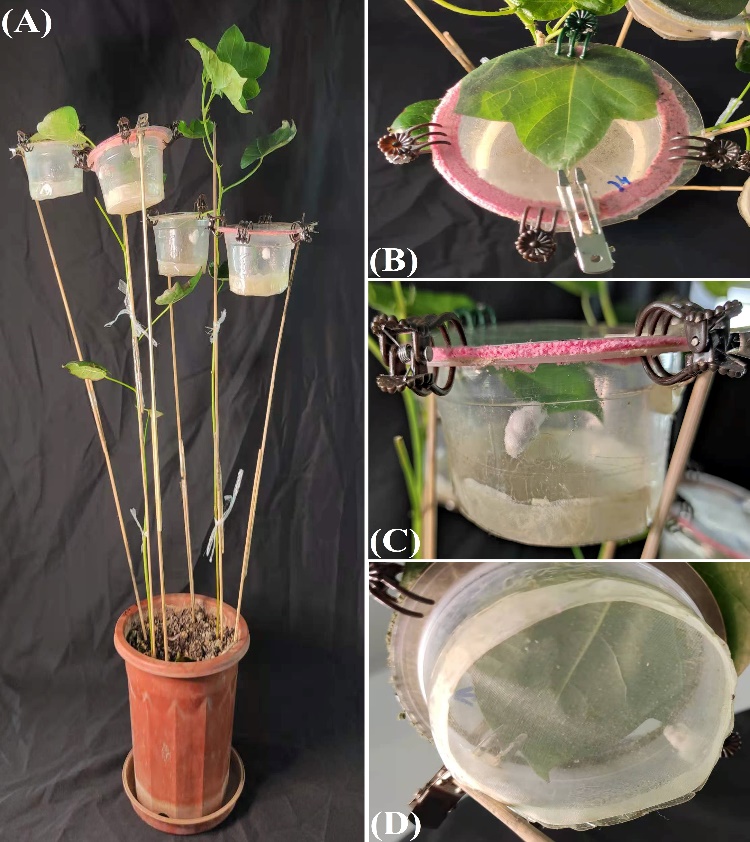


**Figure S1.** Caging of cotton leaves during whitefly rearing and experimentation. **(A)** cotton leaves were caged for whitefly introduction **(B)** fixing of the lid with hair clips to avoid whitefly escape **(C)** Blocking of introduction hole with cotton **(D)** lower view of the sieved bottom of the cage for aeration**.**


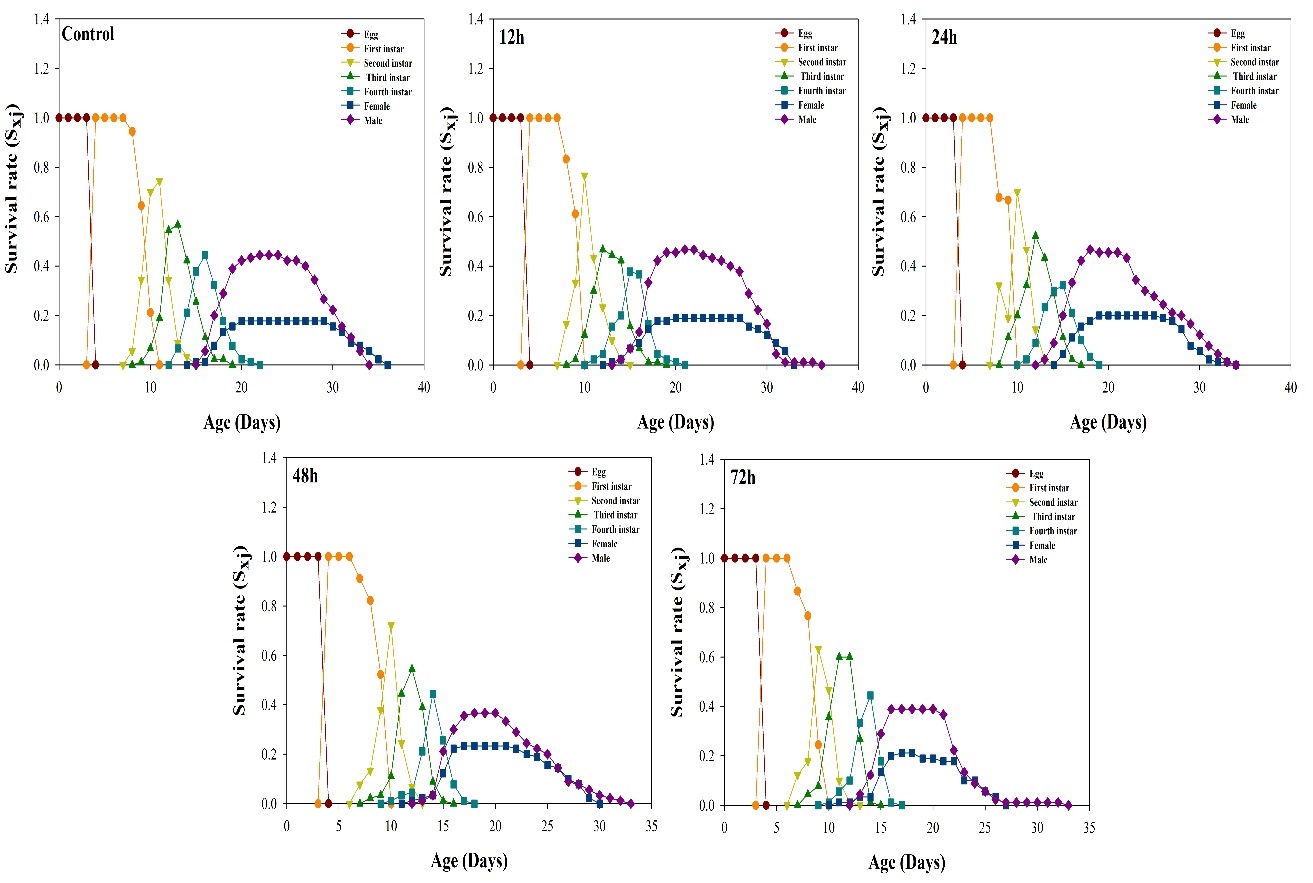


**Figure S2**. Graphs show S*_xj_* (Survival rate of the specific stage) of *B. tabaci* treated at second instar nymph stage exposed to UV-A light for control (0h), 12h, 24h, 48h, and 72h (h=hours).


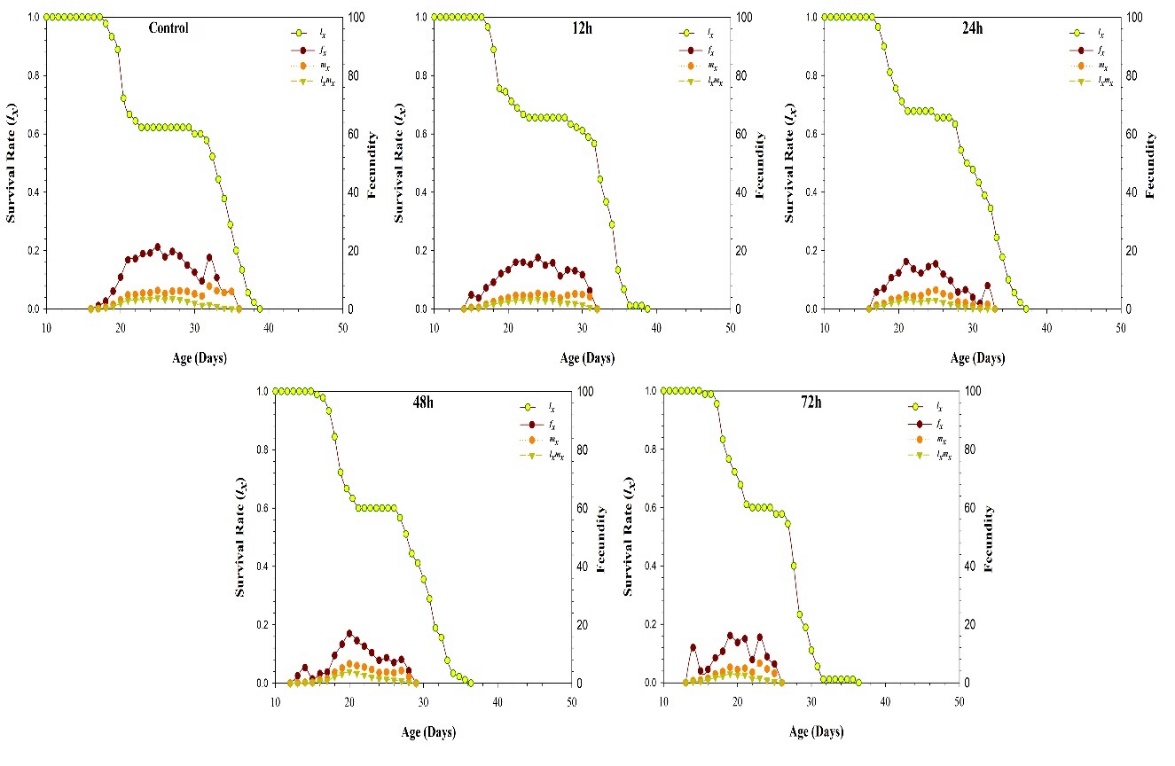


**Figure S3.** Graphs show *l_x_* (Survival rate of the specific stage), *f_x_* (Fecundity of specific age stage), *m_x_*­ (Overall population fecundity), and *l_x_m_x_* (Total maternity) values for *Bemisia. tabaci* nymphs exposed to UV-A light for control (0h), 12h, 24h, 48h and 72h (h=hours).


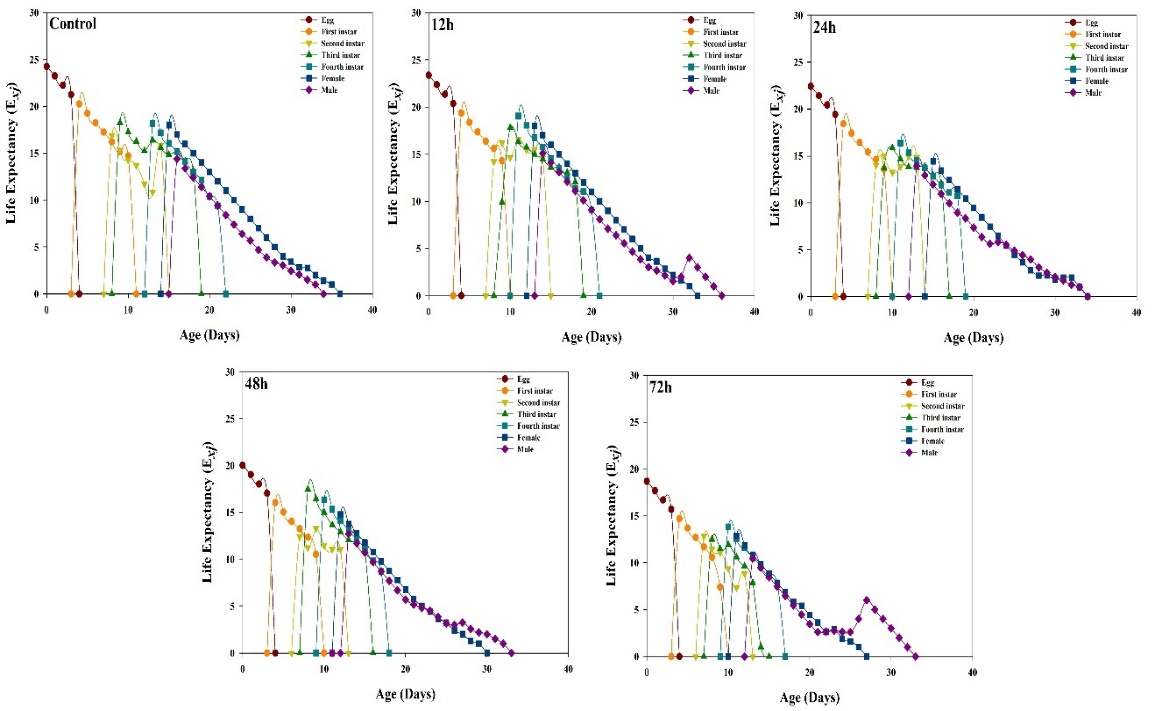


**Figure S4**. Graphs show *E_xj_* (Life expectancy) values of *Bemisia. tabaci* nymphs exposed to UV-A light for control (0h), 12h, 24h, 48h, and 72h (h=hours).


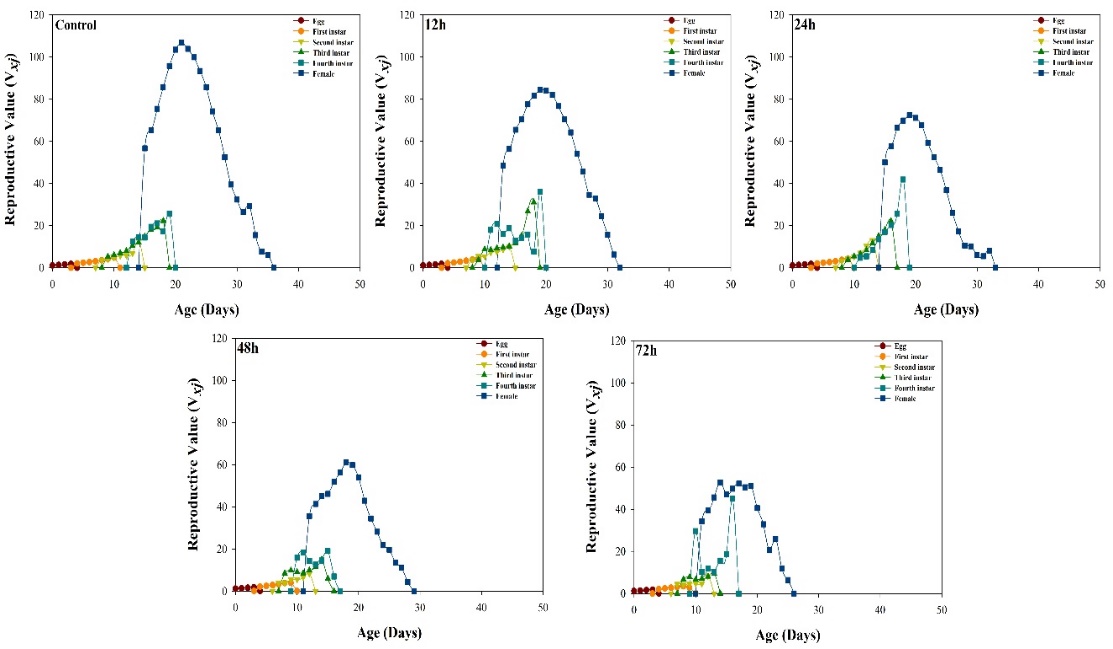


**Figure S5.** Graph show *V_xj_* (Reproduction of a specific stage) values for *Bemisia. tabaci* nymphs exposed to UV-A light for control (0h), 12h, 24h, 48h, and 72h (h=hours).

**TableS1*.*** Effect of UV-A light exposure on the population growth parameters (mean ± SE) of *Bemisia tabaci* adults exposed at the nymphal stage.

| **Treatments** | **Population growth parameters** | | | |
| --- | --- | --- | --- | --- |
|  | **(*r*)** | ***(λ)*** | ***(R0)*** | ***(T)*** |
| Control | 0.14 ± 0.001a | 1.15 ± 0.01a | 38.58 ± 9.11a | 25.56 ± 0.35a |
| 12 hours | 0.15 ± 0.01a | 1.16 ± 0.01a | 34.18 ± 7.66a | 23.49 ± 0.44ab |
| 24 hours | 0.14 ± 0.01a | 1.15 ± 0.01a | 26.90 ± 5.97ab | 23.18 ± 0.30ab |
| 48 hours | 0.15 ± 0.01a | 1.16 ± 0.01a | 24.61 ± 5.21ab | 21.50 ± 0.33b |
| 72 hours | 0.14 ± 0.01a | 1.15 ± 0.01a | 18.42 ± 4.04b | 20.22 ± 0.40c |

The same small letters in the same column are not significantly different as calculated using the paired bootstrap test at the 5% significance level. r = Intrinsic rate of increase day-1; λ = Finite rate of increase day^-1^; R0 = Net reproductive rate (offspring per individual); T = Mean generation time.

***Table S2:*** Percentage mortality of *Bemisia tabaci* exposed to UV-A light for the different durations and treated with different concentrations of *Cordyceps fumosorosea*

|  |  | **Control**±**SE** | **12hours**±**SE** | **24hours**±**SE** | **48hours**±**SE** | **72hours**±**SE** | **Statistics** |
| --- | --- | --- | --- | --- | --- | --- | --- |
| ***Bemisia tabaci* exposed to UV-A light** | **Tween-80** | 0.00±0.00 d | 0.00±0.00 d | 0.00±0.00 d | 0.00±0.00 d | 0.00±0.00 d | - |
|  | **1×10^8^** | 81.49±4.24 a*B* | 95.00±2.89 a*AB* | 95.00±2.89 a*AB* | 94.74±3.04 a*AB* | 100.00±0.00 a*A* | F_4,14_=5.36;*P* <0.05 |
|  | **1×10^7^** | 69.56±2.52 ab*B* | 83.33±4.41 ab*AB* | 83.33±6.01 ab*AB* | 89.47±3.04 ab*AB* | 93.25±4.39 ab*A* | F_4,14_=4.42;*P* <0.05 |
|  | **1×10^6^** | 50.96±3.75 bc*B* | 78.33±4.41 ab*A* | 70.00±5.77 ab*AB* | 82.46±7.65 ab*A* | 82.89±6.32 ab*A* | F_4,14_=5.26;*P* <0.05 |
|  | **1×10^5^** | 49.39±8.13 bc*A* | 65.00±2.89 b*A* | 63.33±8.82 bc*A* | 61.40±9.28 bc*A* | 72.81±6.18 bc*A* | F_4,14_=1.29;*P* >0.05 |
|  | **1×10^4^** | 35.53±4.76 c*A* | 35.00±7.64 c*A* | 40.00±7.64 c*A* | 52.63±8.04 c*A* | 54.39±4.39 c*A* | F_4,14_=1.97;*P* >0.05 |
|  |  | F_5,17_=38.7; *P*<0.01 | F_5,17_=66.9; *P* <0.01 | F_5,17_=32.9; *P* <0.01 | F_5,17_=31.6; *P* <0.01 | F_5,17_=31.6; *P* <0.01 |  |
| ***Cordyceps fumosorosea* exposed to UV-A light** | **Tween-80** | 0.00±0.00 d | 0.00±0.00 d | 0.00±0.00 d | 0.00±0.00 d | 0.00±0.00 d | - |
|  | **1×10^8^** | 86.23±4.60 aA | 76.32±3.17 aAB | 69.56±2.52 aABC | 63.68±2.46 aBC | 53.24±6.62 aC | F_4,14_=8.88;*P*<0.01 |
|  | **1×10^7^** | 75.79±2.11 aA | 65.96±4.69 aAB | 66.05±2.04 aAB | 51.67±2.26 abBC | 41.23±4.38 abC | F_4,14_=16.5;*P*<0.01 |
|  | **1×10^6^** | 53.51±2.23 bA | 44.04±0.96 bA | 50.79±2.24 bA | 42.98±3.82 bcAB | 32.72±1.14 bcB | F_4,14_=12.1;*P*<0.01 |
|  | **1×10^5^** | 43.07±3.19 bc | 33.68±6.23 bc | 35.53±2.45 c | 39.47±5.48 bc | 29.21±4.20 bc | F_4,14_=1.37;*P*>0.05 |
|  | **1×10^4^** | 32.81±2.07 cAB | 23.60±4.16 cAB | 38.86±3.92 cA | 29.12±5.65 cAB | 18.86±4.32 cdB | F_4,14_=3.51;*P<*0.05 |
|  |  | F_5,17_=127; *P*<0.01 | F_5,17_=50.6; *P*<0.01 | F_5,17_=105; *P*<0.01 | F_5,17_=32.9; *P*<0.01 | F_5,17_=20.1; *P*<0.01 |  |

SE= Standard deviation of the slope; Subscripted digits= degree of freedom. Each value is a mean of three replications. Capital lettering is showing the statistical difference in a concentration exposed to UV-A light for a different duration. Small lettering shows the statistical difference in a single exposure time due to the application of different fungal concentrations. Similar lettering has no statistical significance (*P*<0.05).
